# The Grand Challenge: Integrating Nomothetic and Ideographic Approaches to Human Cognition

**DOI:** 10.3389/fpsyg.2017.00100

**Published:** 2017-01-31

**Authors:** Bernhard Hommel, Lorenza S. Colzato

**Affiliations:** ^1^Cognitive Psychology Unit, Leiden UniversityLeiden, Netherlands; ^2^Leiden Institute for Brain and Cognition, Leiden UniversityLeiden, Netherlands

**Keywords:** research methodology, challenge, Aristotelian mode, Galilean sciences, cognitive sciences

In 1931, Kurt Lewin published a plea for the transition from what he identified as the Aristotelian mode of thought in psychology to a Galilean mode (Lewin, 1931). According to Lewin, the Aristotelian mode is characterized by a category-based, top-down approach to study psychological processes. Psychological concepts/categories are taken from everyday-life observations and they tend to be “valuative” and binary in nature (“normal” vs. “pathological;” “true perception” vs. “illusion”). Explanations for novel phenomena are provided by assigning them to an existing category. The scientific ambition to explain psychological mechanisms is restricted to phenomena that can be observed with a high degree of consistency and replicability, which leads to a focus on mean effects and the neglect of individual or situational variability. Lewin emphasizes that this approach implies an anti-thesis between individuality of events or people on the one hand and lawfulness on the other, with the former falling outside the task of science.

In contrast, the Galilean mode focuses on the mechanism underlying a given observation/phenomenon, without restricting the theoretical or empirical treatment of this mechanism to this particular observation/phenomenon. This mode also tends to resolve binary distinctions and oppositions into gradual transitions, so that the explanation of both “normal” and “pathological” behavior, and of both “true perception” and “illusion,” refers to the same basic mechanisms (e.g., by assuming different degrees of contribution or different parameters being used). This leads to a more bottom-up approach, in which various kinds of phenomena are reconstructed and, thus, explained through the interaction of a number of basic mechanisms and principles, irrespective of the category to which these phenomena are thought to belong. Of interest, this approach reduces the theoretical tension between individuality and lawfulness, as the same mechanisms can, and indeed should be used to explain both regularity and deviation.

Lewin identified some preliminary steps toward the Galilean approach in the psychological sciences, but he argued that there is still a long way to go. Today, 75 years later, we see some progress beyond the state of affairs described by Lewin, but we believe that major challenges remain to be tackled. On the one hand, the interest in mechanistic explanations of psychological phenomena has undoubtedly increased, and neural or mathematical models play an increasing role in the scientific discussion. On the other hand, however, the Aristotelian heritage is still visible in various places. Think of the ongoing popularity of the Sternberg approach to information processing (Sternberg, 1969), e.g., as applied by locus-of-slack analyses of dual-task performance (McCann and Johnston, 1992). The major aim of studies in this tradition is to identify the processing stage underlying an experimental effect, such as dual-task costs that are commonly attributed to the response-selection stage (Pashler, 1994). This amounts to a mere Aristotelian categorization of phenomena, without much effort to unravel the actual mechanism—so that we still fail to understand exactly *how* a response is selected and *why* this process is affected by another task.

Similar tendencies can be observed in the cognitive neurosciences, where the identification of the “neural correlates” (i.e., the brain area/system whose activation correlates with the psychological phenomenon of interest) is commonly taken to be sufficient to “explain” the phenomenon. Think of research on the theory of mind, a fascinating cognitive skill that many authors feel to be sufficiently explained by the fact that activity of the right TPJ systematically correlates with employing it (e.g., Saxe et al., 2004). *How* these systems generate the skill remains a mystery however.

One can think of many reasons why we should try harder to move our science toward a more Galilean approach, and we have discussed one of them elsewhere (Hommel and Colzato, 2015). A particularly important reason relates to the relationship between nomothetic and ideographic approaches (i.e., approaches that seek to establish general laws vs. those that seek to account for individual differences) however. As Lewin points out, the practice of explaining phenomena by categorizing and sorting them into types provides a strong motivation to ignore variability within a given category. This does not only apply to the phenomena assigned to a particular category (which for instance explains the lack of interest in differences between tasks that seem to affect the same processing stage, such as flanker, Stroop, and Simon tasks) but it in particular applies to individual differences. In the cognitive sciences and neurosciences, individual differences are commonly taken to reflect random variability that needs to be reduced as much as possible. Indeed, the widespread use of statistical methods that either control and “average-out” individual differences, such as repeated-measures ANOVAs, or that consider individual differences as noise that works against the actually interesting effect, such as univariate ANOVAs and related procedures, demonstrates that both inter-individual and intra-individual differences are not only irrelevant but may even provide an obstacle for explaining the investigated phenomenon.

We suggest that progress in our discipline demands that we overcome this practice which, following Lewin's reasoning, would push our scientific practice further toward a more mature, truly Galilean mode. The major goal would thus no longer exist in sorting phenomena into categories but rather identify core mechanisms that can be used to reconstruct *both* each given phenomenon *and* individual variability therein. The first step toward understanding a phenomenon would no longer consist in finding an optimal definition that seeks to eliminate the conceptual overlap with other phenomena, so that we would no longer need to argue whether and exactly how, say, emotion is different from motivation and cognition, or how empathy differs from contagion and imitation. Instead, this analytical approach would be replaced by a more synthetic approach (in the sense of Braitenberg, 1984; see Hommel and Colzato, 2015), which consists in identifying the way core mechanisms (described with a degree of specificity that goes beyond just indicating the neural area housing them or mere verbal labeling, as in stage approaches) interact to produce the to-be-explained behavior. Such a practice is not too different from common theorizing in behavioristic approaches, only that the toolbox of available core mechanisms need not be restricted to stimulus-response associations. Rather than identifying information-processing stages that are further treated as black boxes, we would need to move on and explain how combinations of core mechanisms are able to generate the performance currently ascribed to each of these stages. Most importantly, however, we would no longer try to eliminate individual differences but build mechanistic theories that explain both mean effects and individual variability. In other words, we would try to understand the mechanics of intra- and inter-individual variation and would directly connect these mechanics to the core mechanism being held responsible for the mean effect.

A serious attempt of integrating nomothetic and ideographic approaches to human cognition would not only move our discipline to a more mature scientific level, it would also be likely to make empirical studies more efficient (as both mean effects and individual variability can serve as data) and account for at least some portions of what is commonly considered the replication crisis in psychology. Let us take the relationship between mood and creativity as an example. Numerous studies have suggested that better mood improves creativity, but a recent meta-analysis has also revealed numerous failures to replicate this relationship (Baas et al., 2008). An Aristotelian approach would consider this state of affairs very problematic and a strong reason to raise doubts in the impact of mood on creativity. A Galilean approach would suggest another research strategy however.

First, it would not follow the practice of first categorizing all sorts of performance as “creative” and then seeking for one coherent explanation. Rather, it would try to reconstruct one given behavioral performance by referring to known basic mechanisms, which in turn would quickly reveal that some “creativity tasks” rely on converging mechanisms while others rely on diverging mechanisms, that some of these tasks rely more on the verbal vocabulary and verbal intelligence than others, and so forth. From these insights into differences regarding the core mechanisms a systematic categorization of tasks may emerge, but the resulting category system would be a result of understanding the underlying mechanisms rather than the vanishing point of seeking for them. And it would be motivated by scientific insight rather than by the questionable linguistic analyses of fuzzy everyday concepts.

Second, a Galilean approach would have the ambition to explain and predict intra- and inter-individual variability. How this ambition can prevent the misinterpretation of failures to replicate can be illustrated by means of two studies from our lab. Based on previous speculations that creativity may be related to dopamine, Chermahini and Hommel (2010) studied the relationship between performance in two creativity tasks (one tapping into convergent and the other into divergent thinking) and the spontaneous eyeblink rate, a clinical indicator of the individual dopamine level in the nigrostriatal pathway. Two outcomes are important for our purposes: (A) Performance in divergent-thinking task was predicted by eyeblink rates while performance in the convergent-thinking task was not, which reinforces the suspicion that there is no such a thing as unitary “creativity;” (B) the relationship between performance in the divergent-thinking task and the indicated dopamine level followed an inverted U-shaped (as shown in Figure 1), so that a medium level was associated with the best performance. Note that the first outcome suggests that seeking for a relationship between mood and “creativity” may be moot (as no task is likely to capture creativity as a whole), while important relationships between mood and some mechanisms underlying creative performance may truly exist.

**Figure 1:**
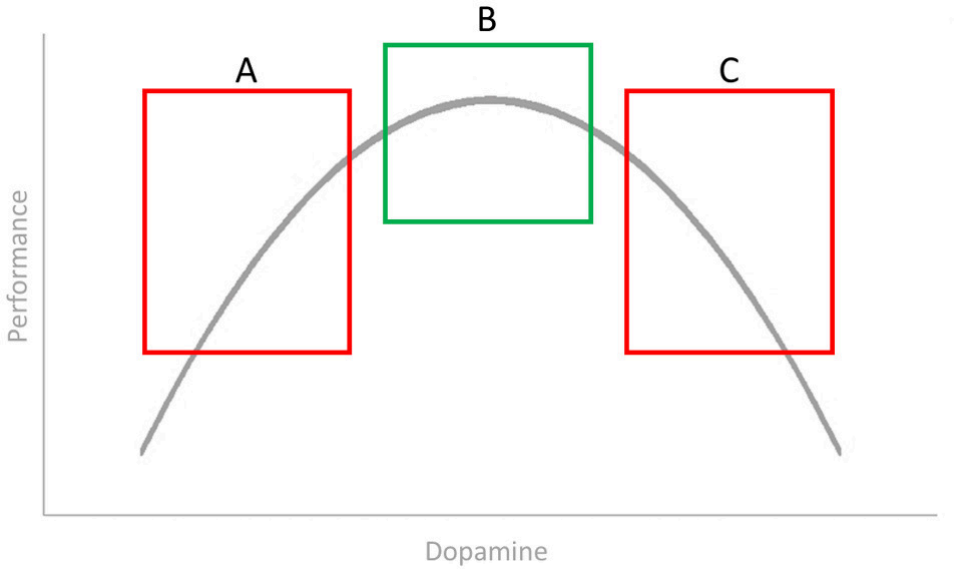
**Performance in divergent thinking as a function of the spontaneous eyeblink rate (idealized)**.

Also note that the demonstrated non-linear relationship may easily account for quite a number of non-replications. As mood is related to striatal dopamine levels, one would expect that individuals having a low level (those falling into the A box in the figure) are likely to benefit from a mood-induced increase of the dopamine level, while individuals with a medium level may not show any effect and individuals with a high level may in fact show impairments. Indeed, Akbari Chermahini and Hommel (2012) reported that low blinkers benefit from positive-mood induction while medium blinkers do not.

It is easy to see that the failure of taking into account individual differences is likely to produce null-effects but that such effects do not at all undermine the claim of a strong connection between creativity and mood. In fact, a Galilean approach to both the conceptualization of the problem and the statistical analysis is likely to have revealed considerable evidence even from studies that are currently considered as failures to replicate. In another study, we have provided evidence that cognitive transfer is mediated by dopamine-related genetic differences, such that individuals with one genetic setup to benefit from cognitive training while others do not (Colzato et al., 2014). Among other things, this raises questions regarding the general validity of claims that cognitive training does not show any transfer effect (Owen et al., 2010), especially if they are based on studies that are silent with regard to the underlying core mechanisms and the role of individual variability (Colzato and Hommel, 2016).

A concerted action to speed up a transition to a Galilean mode does not only require considerable readjustments of our theorizing and empirical practice, it also requires changes in the mindsets of reviewers and readers. We should no longer applaud theories that aim to provide an exhaustive explanation of a particular task or empirical observation but rather learn to value theoretical frameworks that track down core mechanisms in as many phenomena as possible. We should also no longer applaud the unconstrained invention of new concepts and phenomena but rather learn to value parsimonious (in Occam's sense) explanations that can do with already understood basic mechanisms as much as possible. Most importantly, we should no longer be satisfied with models that are restricted to predicting mean effects but increasingly require frameworks that explain both mean effects and individual variation in terms of the same mechanistic insight.

## Author contributions

Authors BH and LC wrote and approved the manuscript.

## Funding

The preparation of this work was supported by an Advanced Grant of the European Research Council (#694722 Metacontrol).

### Conflict of interest statement

The authors declare that the research was conducted in the absence of any commercial or financial relationships that could be construed as a potential conflict of interest.
